# Regional Extinctions and Quaternary Shifts in the Geographic Range of *Lestodelphys halli*, the Southernmost Living Marsupial: Clues for Its Conservation

**DOI:** 10.1371/journal.pone.0132130

**Published:** 2015-07-23

**Authors:** Anahí E. Formoso, Gabriel M. Martin, Pablo Teta, Aníbal E. Carbajo, Daniel E. Udrizar Sauthier, Ulyses F. J. Pardiñas

**Affiliations:** 1 Instituto de Diversidad y Evolución Austral (CONICET), Puerto Madryn, Chubut, Argentina; 2 Laboratorio de Investigaciones en Evolución y Biodiversidad, Facultad de Ciencias Naturales, sede Esquel, Universidad Nacional de la Patagonia San Juan Bosco, Esquel, Chubut, Argentina; 3 División Mastozoología, Museo Argentino de Ciencias Naturales “Bernardino Rivadavia”, Avenida Ángel Gallardo 470, C1405DJR, Buenos Aires, Argentina; 4 Ecología de Enfermedades Transmitidas por Vectores, Instituto de Investigación e Ingeniería Ambiental, Universidad Nacional de San Martín, San Martín, Buenos Aires, Argentina; 5 Instituto Patagónico Para el Estudio de Ecosistemas Continentales (IPEEC) and Facultad de Ciencias Naturales, sede Puerto Madryn, Universidad Nacional de la Patagonia “San Juan Bosco”, Puerto Madryn, Chubut, Argentina; Penn State University, UNITED STATES

## Abstract

The Patagonian opossum (*Lestodelphys halli*), the southernmost living marsupial, inhabits dry and open environments, mainly in the Patagonian steppe (between ~32°S and ~49°S). Its rich fossil record shows its occurrence further north in Central Argentina during the Quaternary. The paleoenvironmental meaning of the past distribution of *L*. *halli* has been mostly addressed in a subjective framework without an explicit connection with the climatic “space” currently occupied by this animal. Here, we assessed the potential distribution of this species and the changes occurred in its geographic range during late Pleistocene-Holocene times and linked the results obtained with conservation issues. To this end, we generated three potential distribution models with fossil records and three with current ones, using MaxEnt software. These models showed a decrease in the suitable habitat conditions for the species, highlighting a range shift from Central-Eastern to South-Western Argentina. Our results support that the presence of *L*. *halli* in the Pampean region during the Pleistocene-Holocene can be related to precipitation and temperature variables and that its current presence in Patagonia is more related to temperature and dominant soils. The models obtained suggest that the species has been experiencing a reduction in its geographic range since the middle Holocene, a process that is in accordance with a general increase in moisture and temperature in Central Argentina. Considering the findings of our work and the future scenario of global warming projected for Patagonia, we might expect a harsh impact on the distribution range of this opossum in the near future.

## Introduction

The Patagonian opossum, *Lestodelphys halli* [1], is endemic to Argentina and the southernmost living marsupial. Its current range extends from 32.5° S (North of Mendoza Province) to 48.6°S (center of Santa Cruz Province), showing an almost continuous distribution through southern Río Negro and Chubut and Santa Cruz Provinces (40° S to 48.6° S), and including a few and isolated records, widely scattered between 32.5°S and 39.5°S (Mendoza, La Pampa and northern Río Negro Provinces [2–5]). In a phytogeographic context, *L*. *halli* inhabits the Patagonian steppe almost exclusively, although sparse records throughout the Monte desert have been found [2, 3, 6, 7]. Our knowledge on the distribution of this marsupial has greatly increased during the last two decades. For more than 65 years, *L*. *halli* was only known from nine specimens from three localities in Chubut and Santa Cruz Provinces [8] and was considered as one of the most poorly known mammals in the world [6, 8, 9]. In contrast, by the end of the 1990's, this species had been reported in more than a dozen localities [6, 9], mainly recovered from owl pellet analyses [2, 5, 7, 10–12]. These findings changed our perception of this opossum from considering a rare to a moderately common species of the extra-Andean small mammal community. These new records demonstrated that this species had been largely overlooked, probably because of its low capture rate with traditional traps [5, 13].

**Fig 1:**
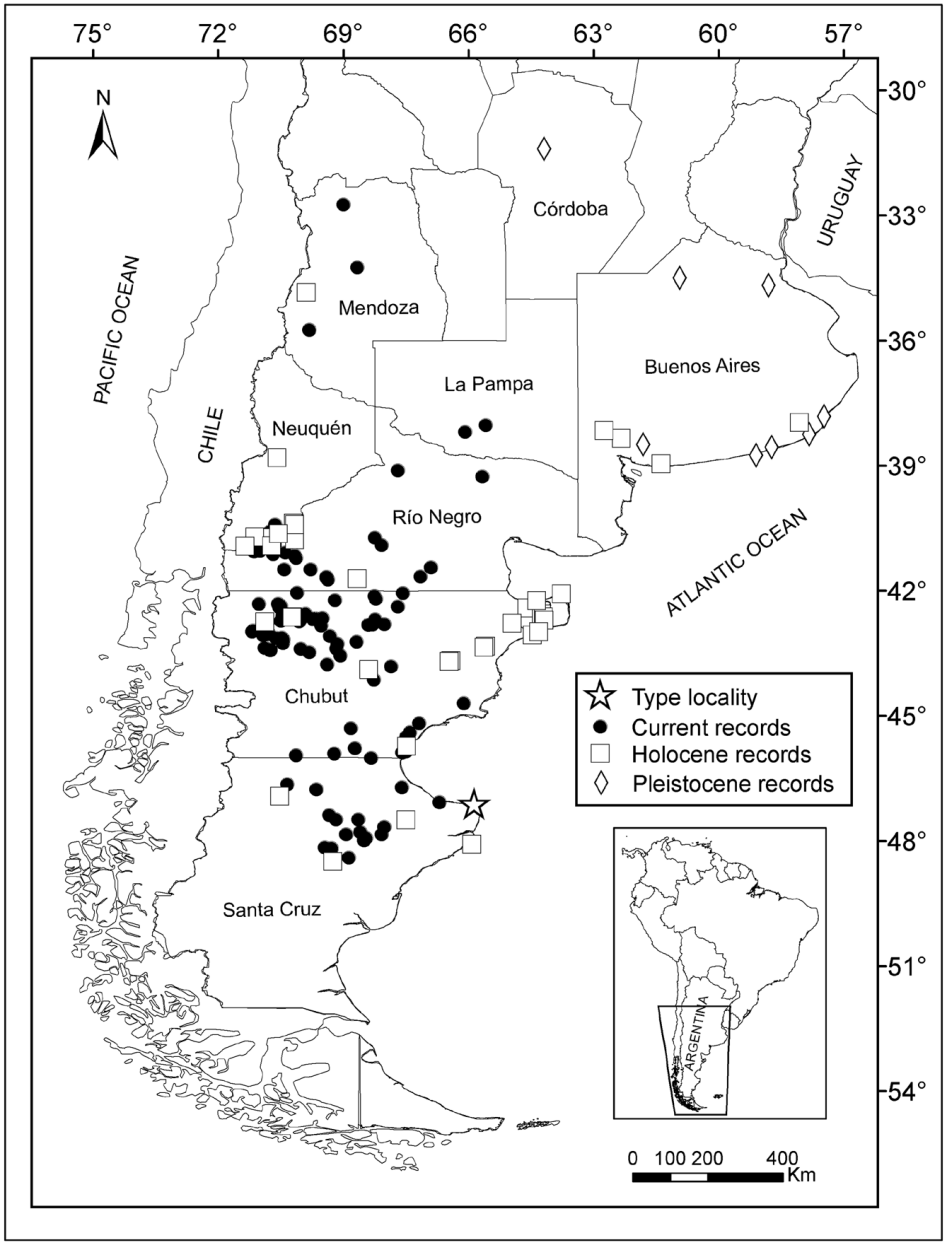
Recording localities for *Lestodelphys halli*.

Contrasting with most living South American marsupials, *Lestodelphys halli* inhabits dry and open environments in southern South America (Fig 1) [5, 14] and also has a rich paleontological record [15–17]. Fossils show that the species lived in most of the Patagonian and Pampean regions during the Quaternary, reaching Central Argentina as far north as 32° S [2, 15, 18–20]. Its extra-limital records have been interpreted as indicators of hostile climatic conditions during the Pleistocene and most part of the Holocene [15, 16, 21–23]. However, the paleoenvironmental meaning of the species' fossil record has been mainly addressed in a subjective framework, without a formal connection to the climatic “space” currently occupied by this animal [15, 16, 24].

The aim of this study was to assess the past and current potential distributions of *L*. *halli* in order to test more accurately their significance as a proxy for cold and dry climatic conditions in the Southern cone of South America. To this end, we identified the most important environmental variables that explain the species’ distribution and inferred the possible causes of regional extinctions and shifts. We also discuss conservation issues, particularly taking into account that the species has been suffering a reduction in its geographic range since the middle Holocene and the future warming that is affecting its range.

## Materials and Methods

The area covered in this study includes the south-central portion of Argentina, from Mendoza (32.5°S), Córdoba and Buenos Aires to central Santa Cruz Provinces (48.6 °S, Fig 1). There are four main ecoregions in this area: Espinal, Pampa, Monte, and Patagonian steppe [25]. The climate in the Espinal ecoregion is warm and wet in the North and warm and dry in the South, with mean annual temperature ranging from 15°C to 20°C and precipitation ranging from 340 mm to 1170 mm [26]. In the Pampa ecoregion, the climate is temperate and sub-humid to humid, with mean annual temperature around 17°C and precipitation ranging from 600 mm to 1200 mm [26], while in the Monte ecoregion, the climate is warm and dry, with mean annual temperature ranging from 10°C to 14°C and precipitation ranging from 100 mm to 200 mm [25]. Finally, in the Patagonian steppe, the climate is temperate cold with strong winds, mainly from the West [27]. The mean annual temperature ranges from 3°C to 12°C and precipitation ranges from 80 mm to 500 mm [28, 29].

The current records of *Lestodelphys halli* used to generate our models were retrieved from trapped specimens, owl pellets, museum specimens and the literature [5, 7, 11, 12]. Trapped specimens and those recovered from owl pellets are housed at Colección de Material de Egagrópilas y Afines “Elio Massoia” (CNP-E) and Colección de Mamíferos (CNP), both from Centro Nacional Patagónico, Puerto Madryn, Chubut, Argentina; Museo Argentino de Ciencias Naturales “Bernardino Rivadavia” (MACN), Ciudad Autónoma de Buenos Aires, Argentina; and Laboratorio de Investigaciones en Evolución y Biodiversidad (LIEB), Universidad Nacional de la Patagonia San Juan Bosco, Esquel, Chubut, Argentina. The paleontological data used to generate our models were based on the fossils collected in the field and records retrieved from the literature [15, 17, 21]. The fossils collected were housed at CNP-E. Permits for collection were given by the Ministerio de Comercio Exterior, Turismo e Inversiones, Subsecretaría de Turismo y Áreas Protegidas de la provincia del Chubut, Argentina (number 209-SSTyAP/08).

Localities in which the species was recorded were divided into “current localities”, which included records from 1921 (when *Lestodelphys halli* was named) to the present, and “fossil localities”, which included records from the Pleistocene (~2.59 million years before present) to the late Holocene (i.e., up to ~200 years before present). As part of the Pleistocene records, we also included those referred to †*Lestodelphys juga* [30], a taxon alternatively considered as valid [2, 21] or suggested as a junior synonym of *L*. *halli* [17]. We accounted for 47 fossil localities (Table 1) and 124 current localities (Table 2). Fossil records included nine from the Pleistocene and early Holocene, 35 from the middle and late Holocene, and three that could not be assigned to any age in particular (i.e., Cueva Tixi, Piedra Museo and Cueva del Manzano-Arroyo Corral; Table 1). These localities belong to the Holocene sensu lato; they were excluded from the models because they lacked an accurate age and could not be allocated to any of the three divisions of the Holocene [15, 18].

**Table 1:** Fossil localities for *Lestodelphys halli*.

Locality number	Locality	Latitude	Longitude	Model	Reference
1	Córdoba	-31.40551	-64.18672	A, B	[30]
2	Junín	-34.50000	-60.93333	A, B	[60]
3	Cueva Grande del Arroyo Feo	-46.93333	-70.51667	A, C	[61]
4	Cueva Sarita 1	-40.92778	-70.71667	A, C	[62]
5	Cueva Sarita 2	-40.92778	-70.71667	A, C	[62]
6	Paraje Paso de los Molles	-40.92778	-70.71667	A, C	[62]
7	Punta Hermengo	-38.25000	-57.83333	A, B	[63]
8	Cueva Tixi	-37.97389	-58.06583	A	[15]
9	Piedra Museo, AEP-1	-47.50000	-67.50000	A	[18]
10	Cueva del Manzano—Arroyo Corral	-40.93333	-71.35000	A	[15]
11	Alero IV del Tromen	-38.81667	-70.58333	A, C	[64]
12	Necochea	-38.55197	-58.72978	A, B	[65]
13	San Martín 1	-38.16667	-62.75000	A, C	[66]
14	Camping Americano	-38.96306	-61.38000	A, B	[21]
15	Napostá Grande	-38.35000	-62.33333	A, C	[52]
16	Cueva Traful	-40.71667	-71.11667	A, C	[49]
17	Astra	-45.73333	-67.48333	A, C	[22]
18	Campo Cerda I	-42.62806	-70.20833	A, C	[22]
19	Cueva Epullán Grande	-40.38917	-70.19444	A, C	[22]
20	Quequén Salado / Indio Rico	-38.74833	-59.10750	A, B	[22]
21	Lle-Cul	-43.33333	-65.58333	A, C	[54]
22	Arroyo Malo 3	-34.85556	-69.88750	A, C	[67]
23	Alero Santo Rosario	-41.71139	-68.66472	A, C	[68]
24	Cueva 4 de La Martita	-48.50000	-69.25000	A, C	[69]
25	Complejo Ferroviario	-37.81583	-57.47556	A, B	[70]
26	Casa de Piedra de Ortega	-40.73333	-70.70000	A, C	[71]
27	Cueva y Paredón Loncomán	-40.78333	-70.16667	A, C	[71]
28	Bajo San Jose	-38.49276	-61.81006	A, B	[72]
29	Punta Medanosa 3	-48.08333	-65.91667	A, C	[19]
30	Rincón Chico 2	-40.41667	-70.16667	A, C	[19]
31	1 km E Riacho San José	-42.41667	-64.60000	A, C	[20]
32	2 km SE Puerto Pirámides	-42.56667	-64.25000	A, C	[20]
33	5 km E Puerto Madryn	-42.78333	-64.95000	A, C	[20]
34	5 km SE Playa Pardelas	-42.63333	-64.20000	A, C	[20]
35	Cueva Caolinera Dique Ameghino	-43.68000	-66.42389	A, C	[20]
36	Cueva de la Virgen	-43.70278	-66.46222	A, C	[20]
37	Cueva Watkins	-42.75028	-70.87361	A, C	[20]
38	Perfil Las Bardas	-43.35678	-65.62667	A, C	[20]
39	Perfil Los Altares	-43.89311	-68.38925	A, C	[20]
40	Piedra Parada I	-42.63722	-70.22361	A, C	[20]
41	Merlo, Buenos Aires	-34.67639	-58.80528	A, B	[17]
42	Ea. San Pablo	-42.70839	-64.18258	A, C	This work
43	Punta Buenos Aires	-42.24228	-64.36047	A, C	This work
44	Punta León	-43.06667	-64.46667	A, C	This work
45	Punta Ninfas	-42.98500	-64.31472	A, C	This work
46	Punta Norte	-42.07588	-63.76928	A, C	This work
47	La Marcelina 1	-40.64000	-70.54806	A, C	This work

**Table 2:** Current localities for *Lestodelphys halli*.

Locality number	Locality	Latitude	Longitude	Model	Reference
1	Cabo Tres Puntas	-47.10000	-65.86	A	[1]
2	Pico Salamanca	-45.40000	-67.40000	A	[73]
3	Ea. Los Manantiales	-43.40000	-70.01667	A	[74]
4	Choele Choel	-39.26667	-65.66667	A, B, C	[75]
5	2 km NNW RN 40 y RP 237	-40.41667	-70.63333	A, B, C	Pearson O. Field notes 1982–1983: 79 (MVZ Library)
6	Cerro Leones	-41.06667	-71.13333	A, B, C	[76]
7	Cañadón Las Coloradas, Alm. El Manzano	-40.65000	-70.78333	A, B, C	[77]
8	Pampa de Nestares	-40.58333	-70.75000	A, B, C	[78]
9	Cerro Castillo (= Guacho), Paso Flores	-40.58333	-70.66667	A, B, C	[79]
10	10 km E Clemente Onelli	-41.16667	-70.16667	A, B, C	[6]
11	10 km WSW Comallo	-41.50000	-70.41000	A, B, C	[6]
12	15 km SE Los Menucos	-40.91667	-68.08333	A, B, C	[6]
13	Chacras de Coria	-32.75000	-69.00000	A, B, C	[6]
14	Ea. Tehuel Malal	-41.03333	-71.16667	A, B, C	[6]
15	Meseta El Pedrero	-46.77283	-69.64150	A, B, C	[6]
16	Parque Nacional Lihuel Calel	-38.03333	-65.58333	A, B, C	[6]
17	30 km NW Pampa de Agnia	-43.47944	-69.81806	A, B, C	[6]
18	Ea. La Gloria	-42.66667	-69.50000	A, B, C	[80]
19	Paso del Sapo	-42.68528	-69.72278	A, B, C	[81]
20	Ea. Yuquiche	-41.50000	-69.78333	A, B, C	[22]
21	Ea. El Gauchito	-45.18333	-67.18333	A, B	[54]
22	Ea. Calcatreo	-41.70000	-69.40000	A, B	[82]
23	Sierra de Talagapa	-42.20000	-68.21667	A, B	[83]
24	Arroyo Mayoco I	-42.75167	-70.87000	A, B	[12]
25	Arroyo Mayoco II	-42.78333	-70.81667	A, B	[12]
26	Arroyo Mayoco III	-42.71667	-70.83333	A, B	[12]
27	Boquete Nahuel Pan	-42.96556	-71.15667	A, B	[12]
28	Cabaña Arroyo Pescado	-43.07375	-70.91358	A, B	[12]
29	Cañadón de la Buitrera	-42.64944	-70.10333	A, B	[12]
30	Cueva Watkins	-42.75028	-70.87361	A, B	[12]
31	Gualjaina	-42.70000	-70.46667	A, B	[12]
32	Nahuel Pan	-42.98750	-71.18306	A, B	[12]
33	Piedra Parada N° 1	-42.65889	-70.10944	A, B	[12]
34	Rio Gualjaina, 1 km W RP 25 y 14	-43.01667	-70.79667	A, B	[12]
35	Ea. Maquinchao, Puesto de Hornos	-41.70000	-68.65000	A, B	[84]
36	Ea. Pilcañeu	-41.13333	-70.68333	A, B	[84]
37	Ea. San Pedro	-42.06667	-67.56667	A, B	[84]
38	Estación Perito Moreno	-41.05000	-71.00000	A, B	[84]
39	Los Altares	-43.84444	-68.42222	A, B	[84]
40	Paso de Los Molles	-40.90000	-70.71667	A, B	[84]
41	Cañadón del Loro	-42.56056	-69.89944	A, B	[10]
42	Colan Conhué	-43.13519	-70.46900	A, B	[10]
43	Paso del Sapo N° 2	-42.68167	-69.66367	A, B	[10]
44	Piedra Parada N° 2	-42.67133	-70.08706	A, B	[10]
45	Cañadon Fuquelén	-40.66667	-70.41667	A, B	[71]
46	Caverna de Las Brujas	-35.75000	-69.81667	A, B	[85]
47	50 km N San Rafael	-34.25000	-68.66667	A, B	[86]
48	Astra	-45.73333	-67.48333	A, B	[86]
49	14 km SE Comodoro Rivadavia	-45.88333	-67.58333	A, B	[87]
50	2 km NW de Gastre	-42.23333	-69.20000	A, B	[11]
51	Cañadon arroyo Quetrequile	-41.69694	-69.40361	A, B	[11]
52	Cañadón Carbón 4	-43.82417	-67.85111	A, B	[11]
53	Cañadón del Painemil	-41.74139	-69.36806	A, B	[11]
54	Cerro Corona	-41.45000	-66.90000	A, B	[11]
55	RP 12, 1 km S Campo de Rueda	-43.09167	-69.32306	A, B	[11]
56	Est. El Torito	-43.27639	-69.14139	A, B	[11]
57	Fofo Cahuel	-42.37536	-70.49417	A, B	[11]
58	Puesto Machín	-41.67778	-69.40139	A, B	[11]
59	Subida del Naciente	-41.66667	-67.15000	A, B	[11]
60	Campo de Cretón	-42.69556	-70.02583	A, B	[11]
61	Campo de Netchovitch	-42.32528	-70.55833	A, B	[11]
62	RP 12 cercanías Cerro Cóndor	-43.38889	-69.17028	A, B	[11]
63	Confluencia ríos Gualjaina y Chubut	-42.60361	-70.37458	A, B	[2]
64	Comodoro Rivadavia	-45.86667	-67.50000	A, B	[7]
65	Ea. La Primavera	-47.85137	-68.93416	A, B	[7]
66	Laguna La Amarga	-38.20000	-66.08333	A, B	[4]
67	10 km N intersección RP 12 y RP 75	-47.79214	-68.59422	A, B	[5]
68	11 km W Laguna Aleusco	-43.14000	-70.60750	A, B	[5]
69	12.8 km NE intersección RN 40 y RP 17	-43.43000	-70.75028	A, B	[5]
70	13 km SW Holdich	-46.01417	-68.32861	A, B	[5]
71	13.5 km SE Paso del Sapo, sobre RP 12	-42.83917	-69.53361	A, B	[5]
72	16 km NE Los Adobes, sobre RP 58	-43.23083	-68.68167	A, B	[5]
73	17.3 km N RP 49 sobre RP 12	-47.49147	-68.64169	A, B	[5]
74	2.2 km W casco Ea. El Camaruco	-43.26250	-70.44972	A, B	[5]
75	2.5 km W Laguna Honda	-42.81806	-68.30139	A, B	[5]
76	20 km S Gan Gan, sobre RP 67	-42.69583	-68.23222	A, B	[5]
77	6 km S intersección RP 33 y RP 12	-42.69750	-70.12556	A, B	[5]
78	36 km E Sarmiento	-45.78107	-68.72083	A, B	[5]
79	4 km S Tres Banderas, sobre RP 11[	-42.80833	-68.01556	A, B	[5]
80	6 km SSW casco Ea. Cretón	-42.74389	-70.05500	A, B	[5]
81	8 km W Paso del Sapo	-42.68056	-69.67417	A, B	[5]
82	Barranco de las Almejas, Fofo Cahuel	-42.40000	-70.51667	A, B	[5]
83	Cabaña Arroyo Pescado 2	-43.02528	-70.79278	A, B	[5]
84	Cabaña Arroyo Pescado 3	-43.04194	-70.80083	A, B	[5]
85	Campo Cretón, Piedra Parada	-42.70000	-70.03333	A, B	[5]
86	Campo de Cretón 5	-42.69889	-70.06861	A, B	[5]
87	Campo de Pichiñan	-43.56389	-69.06722	A, B	[5]
88	Cañadón Minerales	-46.72111	-67.59083	A, B	[5]
89	Cerro Dragón	-45.30167	-68.81611	A, B	[5]
90	Cerro El Sombrero	-44.13917	-68.26333	A, B	[5]
91	Cofluencia ríos Lepa y Gualjaina	-42.73083	-70.49417	A, B	[5]
92	Costa del Chubut	-42.60472	-70.45778	A, B	[5]
93	Cueva Loncon	-42.32417	-71.02028	A, B	[5]
94	Ea. Cerro Argentino	-47.49461	-69.17803	A, B	[5]
95	Ea. El Piche	-47.99369	-68.50133	A, B	[5]
96	Ea. La Argentina	-44.70417	-66.11444	A, B	[5]
97	Ea. La Española	-47.38312	-69.33606	A, B	[5]
98	Ea. La María	-48.41011	-68.86994	A, B	[5]
99	Ea. Mallín Grande	-42.38556	-67.69028	A, B	[5]
100	Ea. San José	-48.16728	-69.44400	A, B	[5]
101	Ea. Sierras del Carril	-45.95184	-70.12833	A, B	[5]
102	Ea. Talagapa	-42.13778	-68.25472	A, B	[5]
103	Escuela N°59 Fofo Cahuel	-42.40833	-70.52944	A, B	[5]
104	Est. Los Manantiales	-45.51139	-67.48583	A, B	[5]
105	Proximidades de Salina Grande	-42.05389	-70.10583	A, B	[5]
106	Puesto El Cuero	-48.18367	-69.28033	A, B	[5]
107	Río Pinturas, 7 km aguas abajo confluencia río Deseado	-46.65276	-70.34266	A, B	[5]
108	Laguna Aleusco	-43.17139	-70.43889	A, B	[5] (erroneously referred as *Thylmays pallidior*; reidentified herein)
109	Ea. La Mimosa	-43.37889	-70.88167	A, B	[88]
110	Sierras de Tecka	-43.42861	-70.75000	A, B	[89]
111	Valle de la Luna	-39.12051	-67.68824	A, B	Fabián Llanos pers. com.
112	20 km NW Los Menucos	-40.73531	-68.24433	A, B	[13]
113	37.2 km SW Sarmiento	-45.91183	-69.21239	A, B	[13]
114	8.5 km WNW El Pajarito	-43.77325	-69.38294	A, B	[13]
115	9.5 km NE intersección RN 40 y RP 17	-43.41361	-70.75000	A, B	[13]
116	Barda Esteban	-40.60875	-70.75694	A, B	[13]
117	Carhue Niyeu	-42.82250	-68.39889	A, B	[13]
118	Pampa de los Guanacos	-40.66283	-70.67694	A, B	[13]
119	Monumento Natural Bosques Petrificados 47°40'S; 67°60'W	-47.67167	-68.01972	A, B	[89]
120	Laguna Manantiales	-47.93333	-68.45000	A, B	[89]
121	Las Piedras	-47.85000	-68.08333	A, B	[89]
122	9 km W Clemente Onelli	-41.22000	-70.13000	A, B	This work (MVZ 179173)
123	8 km WSW Comallo	-41.09000	-70.39000	A, B	This work (MVZ 179193)
124	Mazarredo, RP 14, 40 km E cruce RN3	-47.07892	-66.69687	A, B	This work

We generated six potential distribution models using MaxEnt software 3.3.3e version [31]: three with fossil records and three with extant records. Localities used to generate the fossil models were divided into: All-fossils, including all fossil records from the Pleistocene and Holocene; Last Glacial Maximum (LGM), including records from the Pleistocene and early Holocene; and middle to late Holocene (M/L Holocene), including fossil records from 6000 to 200 years before present. Localities used to generate the current models were divided into: All-current, including all known records from the species description in 1921 to 2013; 1950, including records from 1950 to 2013; and 1950–2000, including localities recorded from 1950 to 2000, in strict accordance with the WorldClim environmental layers (see below). Environmental layers used in the generation of these models included three different databases. The first database included 19 bioclimatic variables from the LGM (~21000 years before present). These are biologically meaningful variables derived from monthly average temperature and rainfall values, with a spatial resolution of 2.5 arc-minutes and based on the Paleoclimate Modeling Intercomparison Project Phase II; PMIP_2_, http://pmip2.lsce.ipsl.fr/ [32]. The second database included 19 bioclimatic variables, monthly average minimum and maximum temperatures and monthly total precipitation from the middle Holocene; CCSM4 [33] with a spatial resolution of 30 arc-seconds. The third database included WorldClim variables (1.4 version; www.worldclim.org) for current climate (from 1950 to 2000), with a spatial resolution of 30 arc-seconds [34]. This set comprised monthly average minimum, mean and maximum temperatures, monthly precipitation, altitude and 19 bioclimatic variables.

We added four categorical variables to the analyses of current localities: global vegetation coverage (globcov), land-form, dominant soil (dominant soil type) and parent material (parentmat; i.e., the material from which soil develops). These variables are from the SOTERLAC database [35] and they incorporated landscape and soil information to the analyses which is not contained in the climatic variables.

The following setup was used for all models: logistic output format, 25% of the records used as training data, 1000 iterations, 10000 background points (randomly selected by MaxEnt) and random seed. The logistic output format was used assigning values of probability of presence to the models, with the following colors: 0.5–1 (red), 0.25–0.5 (orange), 0.1–0.25 (yellow), 0.02–0.1 (green) and 0–0.01 (white). The background point values (i.e., 10000) were selected to determine the variables that better explain the known distribution of the species [36]. Variable contributions were analyzed through jackknife tests (training gain, test gain and area under the curve (AUC); [31, 37]). Finally, the model maps were integrated as ascii format into geographic information systems.

## Results

**Fig 2:**
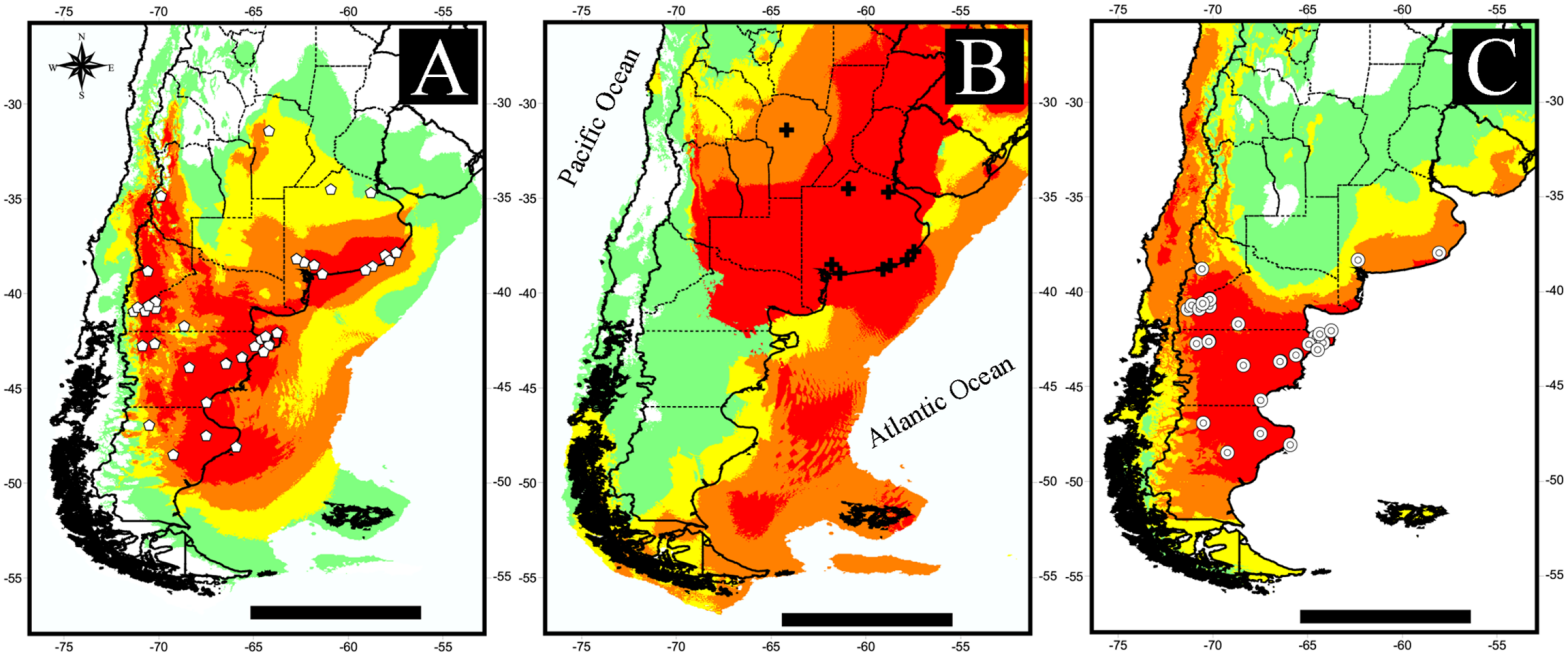
Potential distribution models for *Lestodelphys halli* using fossil records. A) All-fossil, includes all fossil records from the Pleistocene and Holocene; B) LGM, includes records from the Pleistocene and early Holocene; C) Middle to late Holocene (M/L Holocene), includes fossil records up to ~6000 years before present. Scale bar: 1000 km.

**Fig 3:**
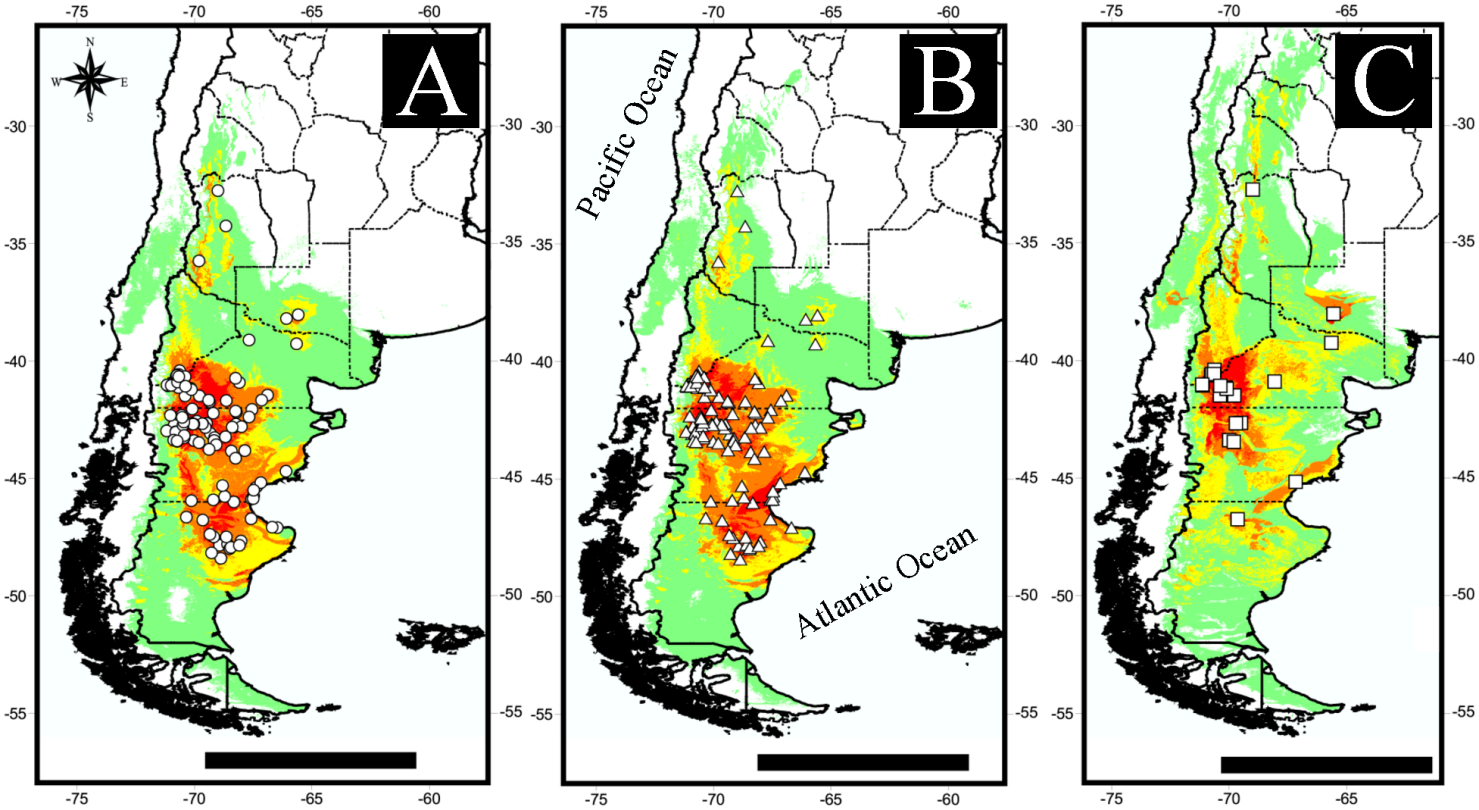
Potential distribution models for *Lestodelphys halli* using current records. A) All current localities, includes all known records since the species description in 1921; B) 1950, includes records after 1950; C) 1950–2000, includes records between 1950 and 2000. Scale bar: 1000 km.

The localities in which *Lestodelphys halli* was recorded are shown in Fig 1 and Tables 1 and 2. The average potential distribution models generated with fossil records are shown in Fig 2. Models showed a decrease in total suitable areas from those including All-fossil records (Fig 2A) to those generated with records from the M/L Holocene (Fig 2C). A contrasting pattern is shown between LGM and the other two models, with a shift in areas with high probability of presence from central-eastern Argentina to a southwestern distribution (Fig 2). Two separated areas of high prediction values appear in the All-fossil model: one along the eastern slope of the Andes from ~32° to 44° S, and the other to the east, from ~41° to 50° S (Fig 2A). The LGM model shows a continuous area of high prediction in central-eastern Argentina, reaching Uruguay and southernmost Brazil (Fig 2B). The M/L Holocene model shows the disappearance of *L*. *halli* from southern Buenos Aires Province (Fig 2C) and a shift to a predominantly Patagonian distribution, similarly to that shown in the models generated with current records (Fig 3). The All-current and 1950 models show two core areas of high probability of presence, one in northwestern Patagonia and the other in southeastern Patagonia (Fig 3A and 3B), joined together by areas with medium (0.25–0.5) and medium-low (0.1–0.25) prediction values. The 1950–2000 model shows a rather different pattern, due to the low number of localities included, and presents a large area of high probability of presence in northwestern Patagonia (Fig 3C).

All models performed better than random with AUC values as follows: All-fossil = 0.994± 0.002; LGM = 0.982 ± 0.014; M/L Holocene = 0.980 ± 0.035; All-current 0.986 ± 0.002; 1950 = 0.985 ± 0.002; and 1950–2000 = 0.980 ± 0.006.

The percent contribution of each variable to the models is presented in Table 3 for fossil records and in Table 4 for current records. For fossils, eight variables (precipitation seasonality, mean temperature of the coldest quarter, precipitation of the driest month, precipitation of the warmest quarter, temperature seasonality, mean temperature of the driest quarter, January average maximum temperature and July average maximum temperature) contributed >40% to each of the models (**boldface** in Table 3). Jackknife tests of variable importance with fossil records using training gain, test gain and AUC on test data recovered different sets of variables (Table 3). For current models, four variables (dominant soil, temperature seasonality, August maximum temperature and precipitation of the warmest quarter) contributed >40% to each of the models (**boldface** in Table 4).

**Table 3:** Percent of contribution of each variable for the three fossil models (All-fossil, LGM and M/L Holocene) generated for *Lestodelphys halli*. Important variables are indicated in boldface.

	All fossil		LGM		M/L Holocene	
Variable	Contribution	Permutation	Contribution	Permutation	Contribution	Permutation
Precipitation seasonality	—	—	**30.5**	3.5	—	—
Mean temperature of coldest quarter	**18.1**	5.1	**28.8**	6.8	—	—
Precipitation of driest month	**17.1**	1.9	—	—	—	—
Precipitation of warmest quarter	**16.5**	1.8	—	—	—	—
Annual mean temperature	13.6	3.9	1.5	9.8	—	—
Temperature seasonality	12.1	13.3	5.3	15.5	**13**	1.4
Minimum temperature of the coldest month	10.2	61.9	1.6	30	10.2	0
Mean temperature of driest quarter	7	5.5	**12**	12.6	2.2	1
Isothermality	2.1	0.2	—	—	—	—
Mean temperature of the warmest quarter	1.1	3.7	—	—	—	—
Maximum temperature of the warmest month	—	—	6	17.9	—	—
Mean temperature of the wettest quarter	—	—	5.3	2.5	5.4	1.3
Precipitation of the coldest quarter	—	—	4.4	0.4	1.4	0.3
Mean diurnal range	—	—	2.9	0	3.5	0
January average maximum temperature	—	—	—	—	**16.9**	5.3
July average maximum temperature	—	—	—	—	**13**	1.6
August average minimum temperature	—	—	—	—	11.6	0
Febrary average maximum temperature	—	—	—	—	9.4	74.5
August average maximum temperature	—	—	—	—	3.1	0.7
Annual precipitation	—	—	—	—	1.7	8.4
Total	97.8		98.3		91.4	
Number of variables with values >1%	9		10		12	
Percentage explained by the three variables with highest values	51.7		71.3		42.9	
*Jackknife test of variable importance*						
Variable with the highest explanatory power	Minimum temperature of the coldest month		Precipitation seasonality		January average minimum temperature	
Variable with the most “unique” information	Temperature seasonality		Precipitation seasonality		Mean diurnal range	
*Jackknife test of variable importance using test gain*						
Variable with the highest explanatory power	Minimum temperature of the coldest month		Precipitation seasonality		No specific variable recovered	
Variable with the most “unique” information	Temperature seasonality		Precipitation seasonality		No specific variable recovered	
*Jackknife test of variable importance using area under curve (AUC)*						
Variable with the highest explanatory power	Minimum temperature of the coldest month		Precipitation of the driest quarter		Temperature seasonality	
Variable with the most “unique” information	Temperature seasonality		Maximum temperature of the warmest month		January average maximum temperature	

**Table 4:** Percent contribution of each variable for the three current models (All current, 1950 and 1950–2000) generated for *Lestodelphys halli*. Important variables are indicated in boldface.

	All current		1950		1950–2000	
Variable	Contribution	Permutation	Contribution	Permutation	Contribution	Permutation
Dominant soil	1.8	0.5	**23.2**	1.5	1.1	0.3
Temperature seasonality	**21.6**	0.5	**15.8**	0.3	**20.8**	0.1
August maximum temperature	**14.1**	0	—	—	**12**	0
Precipitation of warmest quarter	**9.8**	0	1	0.7	**11.8**	2.6
June mean temperature	7.8	0.1	1	0	3	0
July mean temperature	1.1	0.1	—	—	5.8	0.2
Febrary maximum temperature	6.2	0.2	—	—	5	0.5
December precipitation	5.7	74.9	**6.1**	54.5	1.9	81.1
January precipitation	—	—	6.1	0	—	—
Mean temperature of wettest quarter	4.6	0.1	3.9	0	3.9	0.1
Mean temperature of coldest quarter	3.9	0.3	4.7	0	3.5	0.2
Isothermality	2.6	0.1	—	—	2.8	0.4
January maximum temperature	2	0.1	—	—	2.3	0
September mean temperature	1.4	0.1	—	—	1.2	0
Mean temperature of driest quarter	1.1	0.4	—	—	2.3	0.3
August mean temperature	1.1	0.2	2.5	0	2.4	0.1
June maximum temperature	1	0.1	1.4	0	1.9	0
Maximum temperature of warmest month	1	0	—	—	—	—
July maximum temperature	—	—	—	—	4.9	0.1
September minimum tempearature	—	—	4.2	28	—	—
Parental material	—	—	5.2	0.1	—	—
Precipitation seasonality	—	—	3	1.1	—	—
April minimum temperature	—	—	2.8	2.1	—	—
Global vegetation coverage	—	—	2.7	0.2	—	—
Altitude	—	—	2.6	0.4	—	—
June minimum temperature	—	—	1.8	6.2	—	—
Land-form	—	—	1.2	0.2	—	—
August precipitation	—	—	1.1	0.5	—	—
October mean temperature	—	—	1	0	—	—
Total	86.8	77.7	91.3	95.8	86.6	86
Number of variables with values >1%	15		17		17	
Percentage explained by the three variables with highest values	45.5		45.1		44.6	
*Jackknife test of variable importance*						
Variable with the highest explanatory power	Precipitation of warmest quarter		September minimum temperature		August maximum temperature	
Variable with the most “unique” information	Global vegetation coverage		Dominant soil		Global vegetation coverage	
*Jackknife test of variable importance using test gain*						
Variable with the highest explanatory power	Precipitation of warmest quarter		Dominant soil		Precipitation of warmest quarter	
Variable with the most “unique” information	Land-form		Dominant soil		No specific variable recovered	
*Jackknife test of variable importance using area under curve (AUC)*						
Variable with the highest explanatory power	Precipitation of warmest quarter		Isotermality		Precipitation of warmest quarter	
Variable with the most “unique” information	No specific variable recovered		No specific variable recovered		No specific variable recovered	

The lowest AUC values were found in models with the fewest number of localities for the species at different times (i.e., LGM and 1950–2000). The number of localities used also influenced the prediction values for different areas; fewer records generated maps with coarser areas, especially in high (0.5–1.00) to medium (0.25–0.50) prediction values (Figs 2B and 3C). The small number of localities also had an effect on the number of environmental variables used to generate the models, with fewer records “needing” more environmental information to explain the potential distribution of the species. This can be seen in the maps generated from each model, with the one with the smallest number of records (i.e., LGM) showing an over-prediction of high probability areas throughout the potential distribution (Fig 2).

## Discussion

The largest number of current localities (>90% of 124 localities) found for *Lestodelphys halli* were within the Patagonian steppe [2, 5, 6], where cool and dry climatic conditions are dominant [29, 38]. The potential distribution models show that the geographic range of *L*. *halli* has changed from the late Pleistocene to the present day. According to these models we can infer that there was a decrease in suitable habitat conditions for the species, which could be mirroring changes in environmental conditions. Although we did not test biological variables (such as biotic interactions and adaptation), which could be influencing the species’ niche [39], we might expect that the future persistence of this species is threatened, considering the results found in our analyses and the apparent climatic trend.

Our findings support that the presence of *Lestodelphys halli* from the late Pleistocene to the middle Holocene in the Pampean region can be related both to precipitation and temperature variables (e.g., precipitation seasonality, mean temperature of the coldest quarter, precipitation of the driest month, temperature seasonality). However, the models generated with current records show that temperature (e.g., temperature seasonality, August minimum temperature) and dominant soil had a more important contribution. Precipitation of the warmest quarter and temperature seasonality are variables very well represented in both fossil and non-fossil models. Therefore, these variables are the determinants of the distribution of *L*. *halli*, which includes areas with cold and dry weather and pronounced temperature and precipitation seasonality. The presence of *L*. *halli* during the late Pleistocene in Buenos Aires Province was associated with colder and drier climatic conditions, a hypothesis partially supported by the presence of other mammals [23, 40] and by different lines of evidence [15, 23, 40–43]. Contrasting with extinctions in other areas of the Southern Hemisphere [44], it seems that extinctions in the Pampas act from the border toward the center of the distributional range, a phenomenon also seen in some rodents [45]. In this context, populations from northern Mendoza and those scattered in central La Pampa Provinces appear to be more vulnerable to becoming extinct, because these areas have been experiencing more mesic conditions during the last century [46]. A similar result was found by Schiaffini et al. [47] for the Patagonian weasel *Lyncodon patagonicus*, a species that has often been reported to inhabit environmental conditions similar to those inhabited by *L*. *halli*, and used as an indicator of cold and dry climatic conditions [48]. The absence of *L*. *halli* in central and southern Patagonia during the late Pleistocene-early Holocene [18, 22, 49] could also be attributed to physiological constraints. Didelphids are characterized by low basal metabolic rates, high thermal conductance and low body temperatures [50]. Therefore, the climatic conditions of the Late Glacial and Postglacial periods might have been too extreme for *L*. *halli* [43] in southern Patagonia.

During the last 5000 years (middle Holocene to Present), the distribution range of *Lestodelphys halli* has shown a clear shift, from a distribution concentrated in central and eastern Argentina, to a southern and western Patagonian distribution (Figs 2 and 3). The late Holocene distribution of *L*. *halli* suggests an almost complete disappearance of the species from the Pampean region, consistent with changes along this period towards a more mesic and humid climate in central Argentina [15, 51]. Only one record in Napostá Grande was recorded in Buenos Aires Province for the late Holocene [52]. In Patagonia, the species has become extinct from the northeastern area, including several localities in Península Valdes (e.g., Punta Norte, Ea. San Pablo) and in the lower course of the Chubut River (e.g., Cueva Caolinera, Lle Cul), as well as the localities of 1 km E Riacho San José, 5 km E Puerto Madryn, Punta Ninfas and Punta León (Table 3). Furthermore, in southern Patagonia the species has disappeared from the central coast of Santa Cruz Province [19].

The models generated with the current localities are consistent with what is known about the geographic distribution of the species [2, 5]. These models show two large high-prediction areas in Patagonia, one in western Río Negro and northwestern Chubut Provinces, and another mostly restricted to northeastern Santa Cruz Province (Fig 3). In addition, very restricted high- to medium-prediction areas were found scattered surrounding the hypothesized relict records (e.g., those in Mendoza and La Pampa Provinces). Interestingly, despite intensive sampling, no individuals were trapped or recovered from owl pellets outside what we consider relict areas (S1 Fig). We note that in the 1950 (Fig 3B) and 1950–2000 (Fig 3C) models, prediction values around the type locality are medium to low. The specimen collected by T. H. Hall, which O. Thomas used for the original description of *Lestodelphys halli*, was captured around 1920 in Cape Tres Puntas, on the eastern coast of northern Santa Cruz Province (Fig 1). Despite the low prediction values in this area, the species was found 63 km west of the type locality (record 124; Table 2), suggesting that *L*. *halli* is still present near the area where it was collected more than 90 years ago [1, 53].

Two events have shaped the recent distribution of *Lestodelphys halli*. One event is ancient, and through it the species has experienced a shift from the Pampas to the Patagonian steppe and Monte desert. In the other event, during the latest Holocene, the species experienced a retraction in areas of high (0.5–1.00) to medium (0.25–0.5) prediction values throughout Patagonia. The latter shows that this opossum is contracting from a broad Patagonian distribution to core areas in central-northern Patagonia and northern Santa Cruz Province. The models presented in this work suggest that these changes are mostly driven by climatic variables (i.e., precipitation and temperature). These are not minor issues because the regional extinctions of small mammals are a widespread phenomenon in southern South America [45, 54] and have involved several sigmodontine and caviomorph rodent species [22, 55–57].

Climate change is already affecting many natural systems around the world [58]. Temperature increase and changes in precipitation patterns are causing more frequent extreme events, such as floods and droughts. In Latin America, a mean warming of 1 to 6°C is projected for the end of this century. This will trigger the loss of biodiversity, the extinction of several species, a decline in water supply, and a decrease in the yields of very important crops, among others [59]. The geographic distribution of *Lestodelphys halli* is mainly modeled by temperature seasonality, August minimum temperature and dominant soil, and this species has lost more than 150,000 km^2^ in eastern Patagonia during the late Holocene. Considering the findings of our work and that Patagonia is not exempt from adverse climatic changes, we might expect a harsh impact on the distribution range of this opossum in the near future.

The information provided in this work highlights the importance of potential distribution maps as tools to better understand the processes linked to recent regional and local extinctions. This information also allows adding testable data for the use of some species as proxies for climatic conditions, both in the past and the present. Moreover, understanding the processes that are behind this kind of phenomenon is essential to elaborate adequate conservation plans.

## Supporting Information

S1 Fig Localities with absence of *Lestodelphys halli*. Map depicting the absence of *L*. *halli* in owl pellets with more than 90 individuals per sample.
(TIF)
