# Supplementary figures and images for: Regional Extinctions and Quaternary Shifts in the Geographic Range of Lestodelphys halli, the Southernmost Living Marsupial: Clues for Its Conservation
Source: PLoS One. 2015 Jul 23;10(7):e0132130. doi: 10.1371/journal.pone.0132130 (PMC4512715; doi:10.1371/journal.pone.0132130)

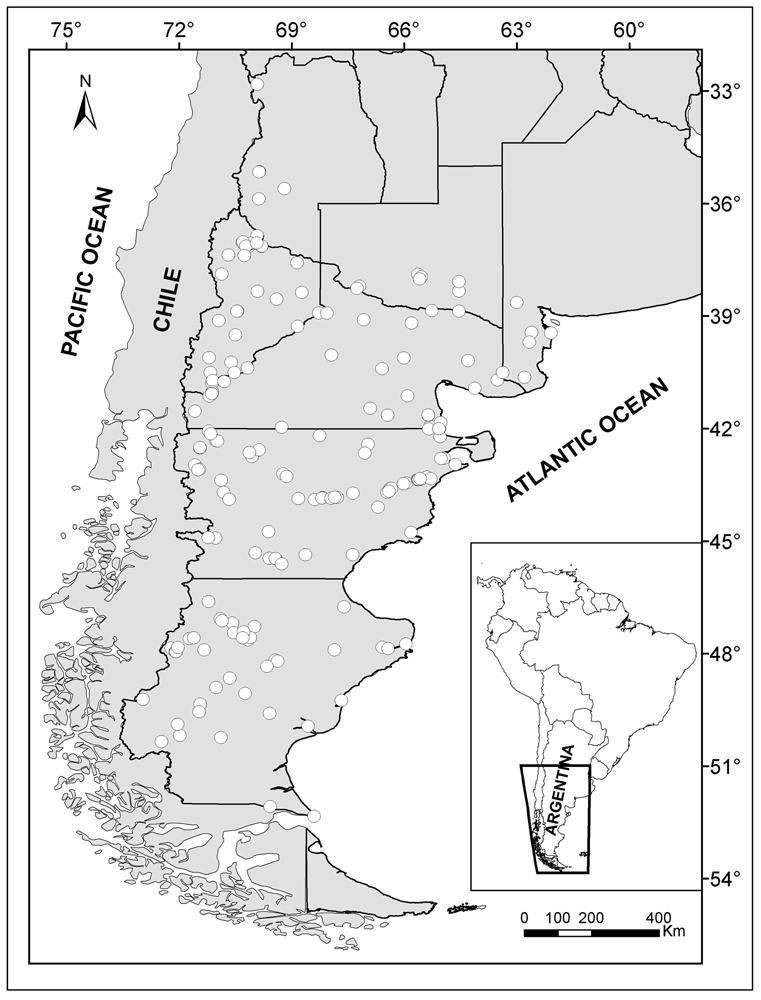

Supplement: S1 Fig — Map depicting the absence of L. halli in owl pellets with more than 90 individuals per sample. (TIF) [file pone.0132130.s001.tif]
